# Isolation and Characterization of Ischemia-Derived Astrocytes (IDAs) with Ability to Transactivate Quiescent Astrocytes

**DOI:** 10.3389/fncel.2016.00139

**Published:** 2016-06-01

**Authors:** Alejandro Villarreal, Gerardo Rosciszewski, Veronica Murta, Vanesa Cadena, Vanina Usach, Martin M. Dodes-Traian, Patricia Setton-Avruj, Luis H. Barbeito, Alberto J. Ramos

**Affiliations:** ^1^Laboratorio de Neuropatología Molecular, Instituto de Biología Celular y Neurociencia “Prof. E. De Robertis”, CONICET, Facultad de Medicina, Universidad de Buenos AiresBuenos Aires, Argentina; ^2^Instituto de Química y Fisicoquímica Biológica, CONICET and Departamento de Química Biológica, Facultad de Farmacia y Bioquímica, Universidad de Buenos AiresBuenos Aires, Argentina; ^3^Institut Pasteur de MontevideoMontevideo, Uruguay

**Keywords:** reactive gliosis, inflammation, ischemia

## Abstract

Reactive gliosis involving activation and proliferation of astrocytes and microglia, is a widespread but largely complex and graded glial response to brain injury. Astroglial population has a previously underestimated high heterogeneity with cells differing in their morphology, gene expression profile, and response to injury. Here, we identified a subset of reactive astrocytes isolated from brain focal ischemic lesions that show several atypical characteristics. Ischemia-derived astrocytes (IDAs) were isolated from early ischemic penumbra and core. IDA did not originate from myeloid precursors, but rather from pre-existing local progenitors. Isolated IDA markedly differ from primary astrocytes, as they proliferate *in vitro* with high cell division rate, show increased migratory ability, have reduced replicative senescence and grow in the presence of macrophages within the limits imposed by the glial scar. Remarkably, IDA produce a conditioned medium that strongly induced activation on quiescent primary astrocytes and potentiated the neuronal death triggered by oxygen-glucose deprivation. When re-implanted into normal rat brains, eGFP-IDA migrated around the injection site and induced focal reactive gliosis. Inhibition of gamma secretases or culture on quiescent primary astrocytes monolayers facilitated IDA differentiation to astrocytes. We propose that IDA represent an undifferentiated, pro-inflammatory, highly replicative and migratory astroglial subtype emerging from the ischemic microenvironment that may contribute to the expansion of reactive gliosis.

**Main Points:**

Ischemia-derived astrocytes (IDA) were isolated from brain ischemic tissue

IDA show reduced replicative senescence, increased cell division and spontaneous migration

IDA potentiate death of oxygen-glucose deprived cortical neurons

IDA propagate reactive gliosis on quiescent astrocytes *in vitro* and *in vivo*

Inhibition of gamma secretases facilitates IDA differentiation to astrocytes

## Introduction

Reactive gliosis is a widespread, however, largely complex phenomenon and its biological role in the induction of neuronal survival or death is still under debate. This is not an on/off mechanism, but rather a graded process from a quiescent state where astrocytes have individual territorial domains to a hypertrophied state with overlapping domains and loss of tissue architecture, ultimately leading to the formation of the glial scar (Burda and Sofroniew, 2014). In addition to the complexity of reactive gliosis as a process, astroglial population has a -previously underestimated- high heterogeneity with cells differing in their morphology, gene expression profile and response to injury (Zhang and Barres, 2010; Wanner et al., 2013; Parmigiani et al., 2015).

Studies on clinical and experimental focal ischemic lesions have shown that brain ischemia invariably produces an ischemic core and a surrounding area of penumbra. The core is a region where all cell types die by necrosis due to the severely limited blood supply in a short period of time, after an episode of acute ischemia. In the hours following ischemia, resident microglia become activated and the core is rapidly invaded by blood-borne immune cells, being neutrophils the first myeloid cell type initially found, followed thereafter by infiltrating monocytes (reviewed in Benakis et al., 2015). The ischemic penumbra surrounding the core is a region where the blood supply was not completely restricted. It remains electrically silent; neurons from this region will die during the next 3–5 days. Penumbral astrocytes become hypertrophied; they change their stellated morphology into a polarized fibrillar reactive phenotype. Days later, reactive astrocytes reorient their processes into a dense mesh that forms a wall-like structure described as the glial scar (Silver and Miller, 2004). Astrocyte scar formation is essentially complete 2–4 weeks after acute insults (Burda and Sofroniew, 2014).

In recent years, it has been clarified that glial response to acute ischemia involves the close interaction of resident glia with the peripheral immune cells (Benakis et al., 2015). Blood brain barrier (BBB) breakdown, chemotactic factors released by activated microglia and damage associated molecular pattern (DAMP) proteins release by necrotic cells, cause the recruitment of professional immune cells from the periphery to invade the ischemic region. It is still not clear how astrocytes interact with invading leukocytes, but *in vitro* experimentation has shown that astrocytes rapidly retract from the leucocytes-invaded area and form scar-like structures (Wanner et al., 2008, 2013; Cregg et al., 2014).

Evidence of the heterogeinity of astroglial cell population has already been reported. For example, an atypical type of astrocyte named aberrant astrocyte (AbA) has been purified from primary spinal cord cultures of symptomatic transgenic rats expressing the SOD1^G93A^ mutation that leads to ALS-like pathology in rodents (Díaz-Amarilla et al., 2011). These AbA cells have a marked proliferative capacity, lack of replicative senescence, secrete soluble factors that induce motor neuron death and seem to derive from a microglia-astroglia phenotypic transition (Trias et al., 2013). It has been also reported that NG2-positive oligodendrocyte precursors (NG2-OPC) migrate toward injury sites (Hughes et al., 2013) and NG2-expressing microglia has been isolated from stab-injury lesions in wild type adult rats (Yokoyama et al., 2006). While typical NG2-OPC can give rise to oligodendrocyte as shown by lineage tracing through *in vivo* imaging (Hughes et al., 2013); NG2 microglia can be turned into a multipotent phenotype by exposure to 70% fetal calf serum (FCS) *in vitro* (Yokoyama et al., 2006). In addition, the formation of neurospheres from ischemic tissue in presence of EGF and FGF has been reported (Shimada et al., 2012). Taken together, all these findings indicate that undifferentiated and/or multipotent local astroglial cell precursors emerge or are expanded in CNS lesions, however, until now their amplification requires extensive genetic or chemical manipulation.

Based on the reported evidence of astroglial heterogeneity in different models of injury, we have here attempted the *ex vivo* isolation of reactive astrocytes from focal ischemic tissue obtained from the rat cerebral cortex, with the aim of finding a sub-population of astrocytes with the ability to propagate reactive gliosis. Taking into account that brain ischemia affects the integrity of the BBB and induce brain cytokines production that create a permissive environment for the recruitment of bone-marrow derived immune cells (Mildner et al., 2007; Benakis et al., 2015), we also investigated whether these astroglial sub-population could be originated from myeloid precursors. Our results show that ischemia-derived astrocytes (IDAs) obtained from early ischemic lesions exhibit atypical phenotypic features, including low replicative senescence, increased cell division and migratory rates, with the potential to induce reactive gliosis on quiescent astrocytes and neurodegeneration on oxygen-glucose deprived neurons. Presumably, the atypical phenotype of these IDA persists due to the presence of local signals that include the activation of Notch1 pathway.

## Materials and Methods

### Materials

Cell culture reagents were obtained from Invitrogen Life Technologies (Carlsbad, CA, USA). Fetal calf serum (FCS) was purchased from Natocor (Córdoba, Argentina). Antibodies were purchased from Chemicon-Millipore (monoclonal anti-RAGE cat# MAB5328; anti-actin cat# MAB1501; anti-NG2 Chondroitin Sulfate Proteoglycan cat# MAB5384); Sigma [monoclonal anti-S100B cat# S2532; polyclonal anti-S100T cat# S2644; monoclonal anti-vimentin cat# V6630; monoclonal anti-glial fibrillary acidic protein (GFAP) cat# G3893; monoclonal anti-Bromodeoxiuridine (5-bromo-2′-deoxyuridine, BrdU)] cat# B8434, Iowa University Hybridoma Bank (monoclonal anti-nestin cat# rat-401) and Dako (polyclonal anti-GFAP Z0334). Bromodeoxyuridine (BrdU), poly-L-lysine, DAPT (*N*-[*N*-(3,5-difluorophenacetyl)-L-alanyl]-*S*-phenylglycine *t*-butyl ester); LPS from *Escherichia coli* O55:B55 and other chemicals were from Sigma. Fluorescent secondary antibodies and peroxidase conjugated secondary antibodies were purchased from Jackson Immunoresearch. Images were taken in an Olympus IX-81 microscope equipped with a DP71 camera or Olympus FV-1000 confocal microscope (Olympus, Tokyo, Japan).

### Cortical Devascularization

Adult male Wistar rats (300–350 g) were obtained from the Animal Facility of the School of Pharmacy and Biochemistry, University of Buenos Aires; the transgenic rat strain [Wistar-TgN(CAG-GFP)184ys] (Mothe et al., 2005) was kindly provided by Dr. Fernando Pitossi (FIL, Buenos Aires, Argentina). Animals were housed in a controlled environment (12/12-h light/dark cycle, controlled humidity and temperature, free access to standard laboratory rat food and water) under the permanent supervision of a professional veterinarian. For all surgical procedures animals were anesthetized with ketamine/xylazine (90/10 mg/kg i.p.). Rats were subjected to a unilateral cortical devascularization (CD) as previously described (Herrera and Robertson, 1989; Villarreal et al., 2011) making every effort to reduce the suffering and the number of animals used. Briefly, rats were placed in a stereotaxic apparatus, and a small surface of skull between the coronal suture and the bregma line was removed to expose the underlying vasculature. A 27-gauge needle was used to cut the overlying dura and tear it away from the underlying pia. A sterile cotton swab was then used to tear back the pia and to disrupt the pial blood vessels overlaying the exposed cortex. Immediately, small sterile cotton pieces embedded in sterile saline solution were laid on the cortical surface until all bleeding ceased (usually less than 50 s). All cotton pieces were removed and the incision in the overlying skin was then closed using the temporal muscle and the attached fascia to cover the lesion site. The animals were housed individually to allow recovery after the surgery for 1, 3, 7, and 14 days. Sham animals were prepared by removing the skull as indicated, leaving the vasculature intact. During the surgery and the whole awakening period, body temperature was maintained by means of a heating pad. Rectal temperature was monitored to preclude hypothermia and to maintain 37.5 ± 0.5°C until animals awoke. The animal care for this experimental protocol was in accordance with the NIH guidelines for the Care and Use of Laboratory Animals, the principles presented in the Guidelines for the Use of Animals in Neuroscience Research by the Society for Neuroscience, the ARRIVE guidelines, and was approved by the CICUAL committee of the School of Medicine, University of Buenos Aires.

### Dissociated Cell Culture and Explants from Adult Ischemic Tissue

At different recovery time points [1, 3, 7, or 14 days post-lesion (DPL)], animals were deeply anesthetized with ketamine/xylazine (90/10 mg/kg i.p.) and sacrificed by decapitation. Brains where rapidly removed and placed in Dulbecco’s modified eagle medium (DMEM) under sterile conditions. Meninges where then carefully removed and a very small region of the cortex containing the ischemic area (typically 2 mm × 2 mm × 2 mm) was dissected and placed in fresh DMEM. A similar fragment from an equivalent cortical area in the contralateral hemisphere, or obtained from sham animals, was removed to be processed as control. Immediately after dissection, cortical explants where mechanically dissociated using scissors and forceps and placed in 15 ml Falcon tubes. After removing the DMEM medium, tissue fragments where digested with 0.25% trypsin for 30 min at 37°C. DMEM supplemented with 10% FCS was added to stop digestion, followed by trituration through a Pasteur pipette until tissue clamps where no longer visible. Dissociated tissue was then centrifuged at 1000 RPM for 5 min, the supernatant was removed and pelleted cells where resuspended in complete DMEM containing 10% FCS, 2 mM L-glutamine, 100 μg/ml penicillin-streptomycin. Cell suspension was immediately transferred to culture poly-L-lysine coated dishes or multiwell plates for further culture characterization and experiments.

For intracortical injection of atypical ischemic astrocytes, adult rats of the transgenic strain [Wistar-TgN(CAG-GFP)184ys] weighing 250–300 g were subjected to focal brain ischemia by the CD method (see above). At 3 DPL, ischemic areas were dissected and atypical astrocytes cultures were obtained as stated before. After 20 DIV, confluent cultures were trypsinized, re-seeded in 10 cm diameter cell culture plates and cultured for additional 25 days to obtain a highly enriched atypical ischemic astrocytes culture. Then, cells were dissociated by treatment with 0.05% trypsin for 5 min at 37°C, washed three times and resuspended in DMEM. Transgenic eGFP-IDA were delivered in the right cortex (bregma, +2 mm; lateral, -2.5 mm; ventral, -1 mm) of naïve wild type Wistar rats with a Hamilton syringe. The volume of injection was of 2 μl, infused over a 4 min period of time, with the syringe left in place for another 2 min to minimize reflux. Contralateral hemispheres received only DMEM.

In a different experimental setup we performed culture explants from ischemic and control tissue. Brains were dissected and cortical explants were obtained by cutting cortical ischemic region into four slices of similar size with a sterile blade. Each slice was immediately plated in poly-L-lysine coated dishes with complete DMEM. Co-culture experiments of tissue explants on primary astrocytes were done essentially in the same way, but explants were seeded on top of confluent astrocytes monolayers obtained from neonatal pups as described below (Villarreal et al., 2014).

### Astroglial Cell Culture

Brains from neonatal rat pups (3 days-old) were removed and brain cortices were isolated following the procedure described in Villarreal et al. (2014). When cells reached confluence (8–10 days), they were subjected to shaking at 180 RPM for 24 h at 37°C to detach microglia and oligodendrocytes. Then, cells were washed with pre-warmed supplemented DMEM, incubated for additional 24 h, trypsinized and re-seeded for the experimental procedures in either 12 or 24 wells plates that were maintained in 5% CO_2_ at 37°C in supplemented DMEM. Cultures obtained with this procedure showed more than 95% astrocytes with positive GFAP staining.

### Primary Cortical Neurons

The cortical neuronal cultures were prepared from embryonic day (E) 16 Wistar rats according to Goslin et al. (1988) with minor modifications described in Villarreal et al. (2011). Oxygen-glucose deprivation (OGD) exposure was performed after 7 days *in vitro* (7 DIV) by extracting the culture medium, replacing it with glucose-free Neurobasal previously saturated for 20 min with a gas mixture composed of 0.1% O_2_, 5% CO_2_, and 94.9% N_2_. Neurons were then incubated for 30 min in the hypoxic chamber in the presence of the previously mentioned gas mixture. Then, medium was removed and replaced with Neurobasal supplemented with B27 equilibrated in normoxic conditions.

### Isolation and Injection of eGFP+ Bone Marrow Mononuclear Cells (BMMCs)

Femurs and tibias from adult rats of the transgenic strain [Wistar-TgN(CAG-GFP)184ys] weighing 300–400 g were dissected. The ends of the bones were cut, and the bone marrow was extruded with 4 ml of α MEM plus 10% FCS by using a needle and a syringe. Aspirates were centrifuged through a density gradient (Ficoll-Paque Plus; 1.077 g/ml), for 30 min at 1000 × g. The supernatant and the interface were combined and centrifuged 10 min at 1000 × g as previously described (Usach et al., 2011). Around 15 × 10^6^ cells were obtained from each animal. The bone marrow mononuclear cell (BMMC) fraction contains mesenchymal stem cells, hematopoietic and non-hematopoietic stem and precursor cells (Herzog et al., 2003; Goel et al., 2009). A total of 15–20 × 10^6^ cells were resuspended in 300 μL of medium and applied through the sacra media artery using a 21G hypodermic needle. A group of rats was injected saline solution as a control.

### Immunofluorescence

After 1, 3, 7, or 14 days post-lesion (DPL), animals were deeply anesthetized with ketamine/xylazine (90/10 mg/kg, i.p.) and perfused through the left ventricle as described (Aviles-Reyes et al., 2010). Brains were cryoprotected, snap frozen and coronal 50 μm thick brain sections were cut using a cryostat. Free floating sections were kept in a cryoprotective solution (30% glycerol, 20% ethylene glycol in 0.05 M phosphate buffer) at -20°C until use. Brain sections from control and ischemic animals (1, 3, 7, and 14 DPL) were simultaneously processed in the free floating state as previously described (Aviles-Reyes et al., 2010; Angelo et al., 2014). Primary and secondary antibodies were diluted in a solution with 3% normal goat serum and 1% Triton X-100 in PBS. Isotypic specific secondary antibodies labeled with Alexa 488 or 594 were used and nuclear counterstaining was made with DAPI 0.1 μg/ml. For cell cultures, fixed cells were washed three times with cold PBS and permeabilized with 0.1% Triton X-100. The procedure was then followed as stated for tissue sections, using the indicated dilutions of the primary antibodies with the exception that Triton X-100 was not included neither in the blocking nor in the antibody solution. Epifluorescence images were obtained in an Olympus IX-81 microscope equipped with a DP71 camera (Olympus, Tokyo, Japan) or in a Zeiss Axiophot (Carl Zeiss, Oberkochen, Germany) microscope equipped with a digital camera (Olympus Q5). Confocal images were taken in an Olympus FV-1000 confocal microscope and Z-stack projections are shown unless stated otherwise in the figure.

### Proliferation Assay

For proliferation rates comparison between primary glia and atypical ischemic astrocytes, BrdU 600 ng/ml was added to the tissue culture medium and cells were fixed 24 h later. Cells where then incubated in 0.5 N HCl in PBS during 30 min followed by incubation in borate buffer 0.1 M for additional 5 min. After three washes with PBS, BrdU incorporation was detected following the immunofluorescence protocol with a specific antibody against BrdU.

### Wound Healing Assay

Primary astrocytes or atypical ischemic astrocytes were cultured until confluence (7–10 days) as stated above. A scratch was made with a sterile micropipette tip to create a cell-free area. Then, medium was changed and after 24 or 48 h, cells were fixed and stained with anti-GFAP and DAPI nuclear counter-staining. Images were captured at different time points (0, 6, 24, 48 h).

### Immunoblotting

Cultured astrocytes were homogenized in NP-40 lysis buffer [10 mM Tris (pH 8.0), 150 mM NaCl, 1% Nonidet P-40, 10% glycerol], with a protease inhibitors cocktail (Sigma), PMSF 1 mM and sodium vanadate 10 mM. Total protein quantification was performed using the Lowry assay (Lowry et al., 1951) and corroborated developing membranes with an anti-actin antibody as loading control. Samples with equivalent protein content were separated by SDS-polyacrylamide gel electrophoresis and transferred to a nitrocellulose membrane as previously described (Ramos et al., 2007). Developing of peroxidase activity was performed using ECL substrate for chemiluminescence (Pierce) and captured in X-Ray film in a dark room.

### Quantitative Studies and Statistical Analysis

Changes in astroglial cell morphology, area covered by the cells, cell division rate and colocalization index (Manders’ colocalization index; Nakamura et al., 2007) were evaluated by using the NIH ImageJ software and plugins on cells or tissue sections immunostained as stated in each figure legend. Each *in vitro* experiment was repeated three times using duplicates or triplicates in each experiment. Experiments involving animals were performed independently three times, and each experiment used at least three animals. Representative experiments and photographs or gels are presented in the figures. The data was analyzed for normal distribution and homogeneity of variances and then subjected to the appropriate test (parametric or non-parametric) as stated in each figure legend. The statistical software package used was GraphPad Prism 5.0 (San Diego, CA, USA) and statistical significance was assumed when *p* < 0.05.

## Results

### Astrocytosis in Brain Ischemia Induced by Cortical Devascularization (CD)

**FIGURE 1:**
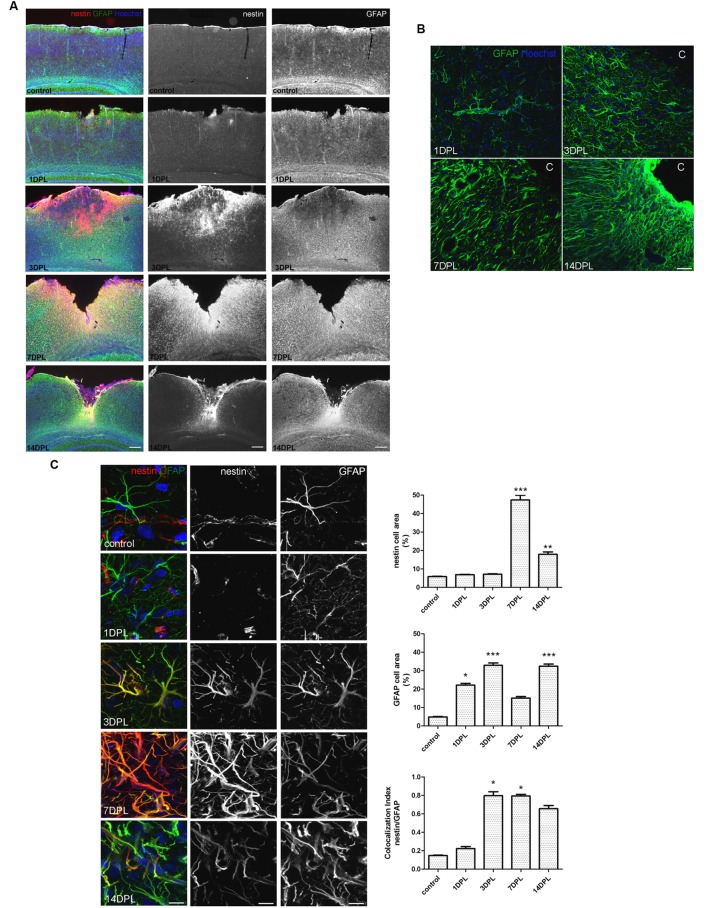
**Focal brain ischemia by cortical devascularization induces nestin expression, GFAP overexpression and glial scar formation in rats. (A)** Low magnification images of nestin (red) and GFAP (green) expression at 1, 3, 7, and 14 DPL (DPL, days post-ischemic lesion). Nestin expression (red) is very low in control and 1 DPL animals and dramatically increased at 3 and 7 DPL, being still present at 14 DPL, bar = 1000 μm. **(B)** Confocal high magnification images show how the astroglial stellated resting phenotype slowly changes toward a reactive, fibrillar and intermingled morphology forming the glial scar by 14 DPL, bar = 30 μm. **(C)** Confocal high magnification images of penumbral astrocytes showing nestin/GFAP colocalization. The initial low nestin immunostaining in the perivascular structures and absence of glial nestin until 1 DPL is dramatically altered by 3 and 7 DPL, showing high nestin expression in penumbral astrocytes. Bar = 15 μm. Quantitative studies performed on tissue sections showed significant changes in nestin cell area that peaked on 7 DPL, while Manders’ colocalization index showed the overlapping expression of nestin and GFAP on the penumbral astrocytes. Because of its calculation, Manders’ overlap coefficient measures the proportion of the signal (nestin) colocalizing with the other signal (GFAP) without taking into account the intensity of the signals. Values are represented as mean ± SEM and statistical significance was confirmed with the non-parametric Kruskal–Wallis test and Dunn’s Multiple Comparison Test. *p < 0.05, **p < 0.01; ***p < 0.001.

Unilateral CD induced a permanent cortical focal ischemia with a clearly defined ischemic core and surrounding penumbra (**Figure 1A**). At 3 days post-ischemic lesion (DPL), the ischemic core was revealed as a GFAP-negative stained area surrounded by intense nestin immunoreactivity (**Figure 1A**). By 7 DPL, penumbral astrocytes surrounding the core showed the change in the classical stellated non-polarized morphology toward a fibrillar phenotype with projections directed to the core (**Figure 1A**). After 14 DPL, astrocytes surrounding the area of the ischemic core have completely lost the symmetrical stellated morphology and reoriented their processes to form the dense mesh typical of the glial scar (**Figure 1A**). High magnification confocal images also show the extreme changes in the phenotype of penumbral GFAP-immunoreactive astrocytes from 1 to 14 DPL (**Figure 1B**). In **Figure 1C** confocal images of nestin/GFAP immunolocalization show that nestin expression, practically absent in the control non-ischemic hemisphere, peaks at 7 DPL in astrocytes, concomitantly with a reduction in GFAP expression levels (**Figure 1C**). Colocalization analysis performed irrespectively of the intensity of the staining, however, showed that nestin expression is mainly related to the GFAP-expressing astrocytes (**Figure 1C**). We conclude that focal brain ischemia by CD induces reactive gliosis with profound changes in the astroglial phenotype, including the expression of the immature glial marker nestin.

### Atypical Astrocytes are Present in Ischemic Tissue

**FIGURE 2:**
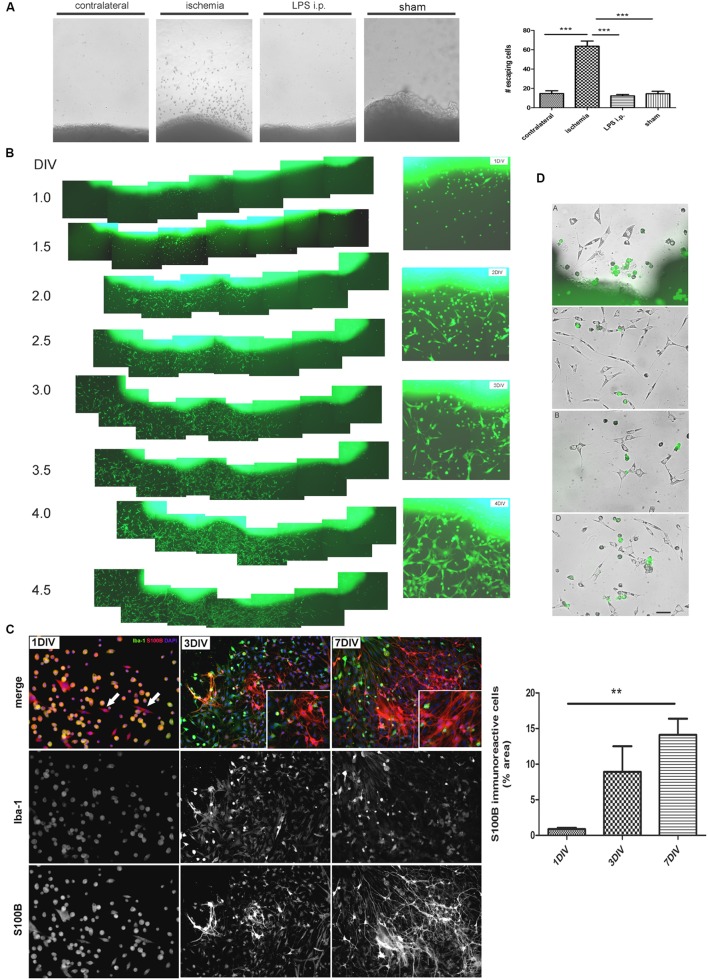
**Ischemia-derived astrocytes (IDAs) are present in ischemic explants and proliferate *in vitro*. (A)** Representative images of cortical explants obtained from 3 DPL ischemic lesions, contralateral hemisphere, LPS-injected animals (5 mg/kg, i.p.) or sham animals showing a large number of cells that spontaneously escape from ischemic tissue. Only cells derived from ischemic explants successfully survive *in vitro*. Quantitative results show the total number of cells escaping from ischemic explants in a 0.5 mm^2^ area sectioned from the microscopic field. Values are represented as mean ± SEM and statistical significance was confirmed with ANOVA and Student Newman Keuls post-test (***p < 0.001). **(B)** Time lapse images acquired every 12 h of the same 3 DPL cortical ischemic explants from eGFP transgenic rat strain [Wistar-TgN(CAG-GFP)184ys] showing the rapid escape of cells from ischemic explants observed from 1 DIV (DIV, days *in vitro*) to 4 DIV. Insets show the detail of different cell morphologies and the abundance of fusiform cells by 4 DIV. Morphology of cells escaping from ischemic explants are different after 1, 2, 3, or 4 DIV. Polygonal or fusiform IDA are the predominant cells after 3–4 DIV. **(C)** Immunocytochemistry images showing the Iba-1 and S100B expression in the cells escaping from ischemic explants. From 1 to 7 DIV the intense Iba-1 expression is reduced and S100B expression increased. Quantitative results show the area occupied by the S100B(+)/Iba-1(-) (S100B-*only*) expressing cells. Values are represented as mean ± SEM and statistical significance was confirmed with the non-parametric Kruskal–Wallis test and Dunn’s Multiple Comparison Test (**p < 0.01). **(D)** Different representative images of IDA escaping away from the 3 DPL ischemic explants after 3 DIV. Rats have previously been injected with eGFP+ BMMC and then were subjected to ischemia by cortical devascularization. Note that eGFP+ cells have not fusiform IDA phenotype. Bar = 15 μm.

In view of the dramatic changes of the astroglial population in the peri-infarct region, we then attempted to isolate the different cell types. For that purpose, we first seeded cortical tissue explants containing the ischemic lesion of 1, 3, or 7 DPL animals, and incubated them on tissue culture plates as described in Section “Materials and Methods.” Soon after platting, cells showing different morphologies escaped and migrated away from the ischemic cortical explants, to a much greater extent compared to explants obtained from LPS-injected animals, sham animals or even isolated from the contralateral non-ischemic cortex (**Figure 2A**). By using ischemic cortical explants from the transgenic eGFP rat strain [Wistar-TgN(CAG-GFP)184ys] and tracking them on a time-lapse microscope, we again observed the profuse cell escape from the ischemic explants (**Figure 2B**). **Figure 2C** shows in detail the different cell phenotypes that escape from the ischemic explants during the first days in culture. Initially, small rounded cells with a macrophage/microglia phenotype, showing Iba-1(+)/S100B(+) immunostaining, escape and coexist with a few polygonal S100B(+)/Iba1(-) cells (**Figure 2C**). Then, extending the time of culture to 3–7 DIV, Iba1(+) cells decrease in number and are segregated from those S100B(+) cells that resulted expanded and still show elongated polygonal morphology (**Figure 2C**). After 7 DIV these elongated polygonal S100B(+) cells increase their number dramatically, densely covering the periphery of the tissue explants as well as distal regions (**Figure 2C**). Quantitative analysis of the area occupied by S100B-only cells showed the significant increase from 1 to 7 DIV (**Figure 2C**). Low GFAP expression was normally associated with the S100B-immunoreactive elongated polygonal cells (**Supplementary Figure S1A**). We conclude that an atypical glial cell type expressing S100B and low GFAP levels spontaneously escapes from ischemic tissue explants and, based on the immunocytochemical profile we named them IDAs.

We have thus shown that IDA are present in ischemic tissue and spontaneous migration occurs in the presence of macrophages/microglia. Knowing that brain ischemia induces a permissive environment for the engraftment of bone marrow-derived myeloid precursors and AbAs obtained from primary spinal cord cultures of symptomatic transgenic rats expressing the SOD1^G93A^ mutation were shown to derive from a microglial-astroglial phenotypical transition (Trias et al., 2013), in the following experiments we analyzed if IDA could derive from myeloid precursors recruited to the ischemic core. For that purpose, we isolated BMMC from the transgenic eGFP rat strain [Wistar-TgN(CAG-GFP)184ys] and transferred them into wild type Wistar rats that were immediately subjected to ischemia by CD. After 3 DPL, cortical explants from the ischemic area were obtained and cultured for 3 DIV. A detailed observation of the cells escaping from explants showed that eGFP positive cells presented a small rounded morphology, and we could not observe eGFP positive IDA (**Figure 2D**). To further confirm successful distribution of eGFP transferred cells in the receptor animal, we evaluated the presence of eGFP positive cells in blood smears and in liver and brain sections. In all these three types of samples we observed eGFP positive cells (**Supplementary Figure S1B**). We conclude that IDA isolated *in vitro* do not derive from bone marrow myeloid precursors recruited to the ischemic tissue.

### Isolation and Characterization of Ischemia-Derived Astrocytes (IDAs)

**FIGURE 3:**
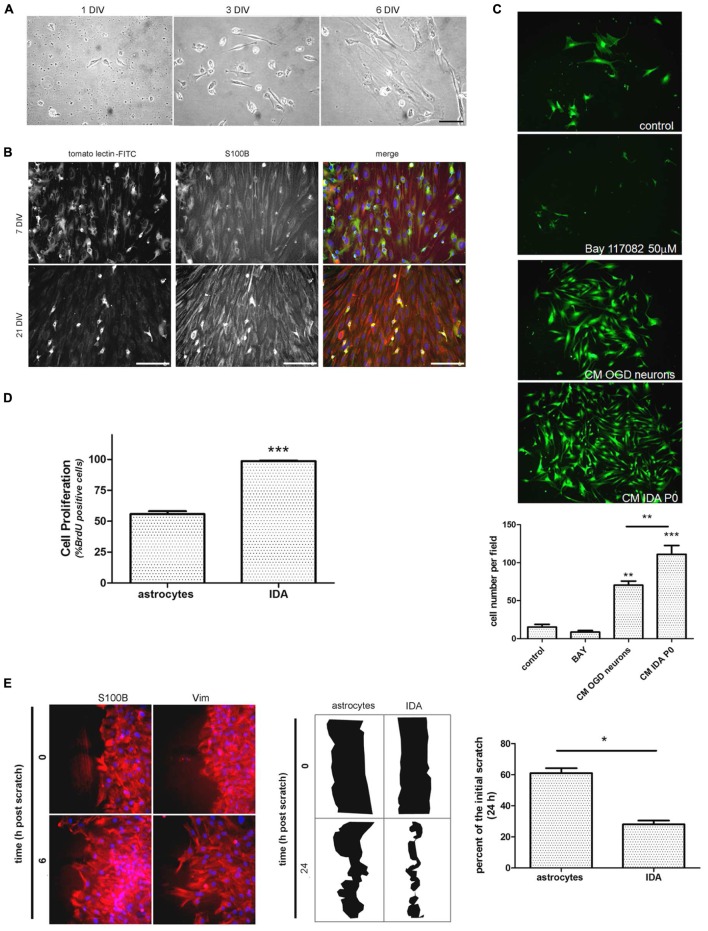
**Ischemia-derived astrocyte can be isolated and amplified *in vitro* showing increased rate of cell division and migration. (A)** Images of the progress of the dissociated culture after 1, 3, and 6 DIV starting from ischemic cortex of 3 DPL animals, bar = 15 μm. **(B)** Representative images of 7 and 21 DIV cultures of IDA stained with the macrophage/microglial marker tomato-lectin FITC (green) and the glial marker S100B (red). Note the reduction in the tomato-lectin staining and the increase in S100B expression as the culture develops. Bar = 20 μm. **(C)** Representative images of low density IDA cultures (7 DIV) derived from eGFP transgenic rats and incubated with conditioned medium (CM) of OGD-exposed neurons, CM of IDA primary culture (P0) or BAY 117082 50 μm. Quantitative results showed the significantly increased IDA yield when the culture was exposed to CM derived from IDA or OGD-exposed neurons. Values are represented as mean ± SEM (cell number per field) and statistical significance was confirmed with ANOVA and Student Newman Keuls post-test (***p < 0.001; **p < 0.05). **(D)** Quantitative analysis of BrdU incorporation after 24 h comparing an IDA culture with quiescent primary cultured astrocytes at the same confluence. Values are represented as mean ± SEM (percentage of BrdU+ cells per field counterstained with nuclear DAPI) and statistical significance was confirmed with the non-parametric Kruskal–Wallis test and Dunn’s Multiple Comparison Test (***p < 0.001). **(E)** Representative images of IDA rapidly invading the artificial wound performed on high density cell culture wounded with scratches, fixed at the indicated times and stained with S100B or vimentin (Vim). Comparative drawings and quantitative analysis of the scratch width after 24 h post-scratch wound injury showing that IDA migrate significantly faster than primary cultured astrocytes. Statistical significance was confirmed by Wilcoxon two tailed *t*-test (*p < 0.05).

After detecting IDA in the ischemic explants, we decided to evaluate whether these cells can be expanded *in vitro* to analyze their cell biology and to compare them with quiescent primary astrocytes. For that purpose, we subjected the animals to brain ischemia and, after 3, 7, or 14 DPL we isolated the cortical ischemic area in the ipsilateral and contralateral hemispheres or in sham animals. The dissociated tissue was cultured to screen the cells yielded after 1 and 7 DIV. While contralateral or sham explants rendered a very reduced number of cells that did not survive *in vitro*, 3 DPL ischemic explants showed a larger total cell number and reduced number of the macrophage/microglia phenotype, with enrichment in IDA (fusiform morphology; S100B^high^/GFAP^low^ immunoreactivity), when analyzed at 7 DIV (**Supplementary Figure S2**). A closer observation of the progression of culture of cells obtained from 3 DPL animals initially showed small rounded cells and few isolated fusiform cells at 1 DIV (**Figure 3A**). Extending the culture to 3 and 6–7 DIV dramatically increased the abundance of fusiform polygonal cells similar to IDA observed escaping from ischemic tissue explants (**Figure 3B**). This latter cell type rapidly propagated, forming islands of cells (3 DIV) that quickly colonized the plate to become the predominant phenotype by 6–7 DIV (**Figure 3B**). Immunofluorescence studies showed that this prevailing fusiform cell type was immunoreactive for S100B and weakly positive for Iba-1, tomato lectin or GFAP (**Figure 3B**) as were IDA obtained from the ischemic explants. Additional studies showed that the initially weak tomato lectin staining at 7 DIV was further reduced by 21 DIV, showing stable S100B and low GFAP immunoreactivity (**Figure 3B**). Extending the time in culture beyond 21 DIV and even increasing the number of passages did not alter significantly IDA morphology, which persisted as polygonal fusiform cells with S100B^high^/GFAP^low^ immunoreactivity without significant changes in morphology or growth (**Figure 3B** and **Supplementary Figure S3**). IDA rapidly populate the entire plate, but we found that this process can be speeded up by incubation with conditioned medium (CM) from OGD-exposed neurons presumably containing DAMPs or by IDA CM itself (**Figure 3C**). This fact, together with the consideration that IDA were isolated from deeply damaged tissue containing a myriad of cytokines and DAMPs, that collectively can activate NF-κB pathway, pointed us to test the effect of NF-κB blockage in IDA initial proliferation. Blockage of NF-κB activity with BAY 117082 failed to significantly decrease IDA abundance but rendered smaller cells (**Figure 3C**). We conclude that IDA obtained from ischemic tissue can be amplified *in vitro*, they show a stable phenotype and S100B^high^/GFAP^low^ immunoreactivity and that they have reduced replicative senescence.

As mentioned above, IDA rapidly populate the cell culture plates and can be propagated for several passages with a stable phenotype. Thus, we decided to study IDA cell division rate and to compare it with that of primary astrocytes obtained from neonatal pups, since primary astrocytes cannot be obtained from non-injured adult brain tissue. Studies were performed on IDA cultured for 7 DIV derived from 3 DPL ischemic brains, and BrdU incorporation was compared to that achieved by a primary astroglial culture of a similar confluence. As predicted, IDA showed an increased BrdU incorporation compared to primary astrocytes (**Figure 3D**) and migrated faster in wound healing scratch assays, rapidly closing the wound in 24 h while primary astrocytes required 48–72 h for a similar effect (**Figure 3E**; Villarreal et al., 2014). We conclude that IDA isolated from ischemic brains are highly proliferative and have increased migratory capacity *in vitro*.

### IDA Secrete Soluble Factors that Induce Reactive Astrogliosis and Neuronal Death

**FIGURE 4:**
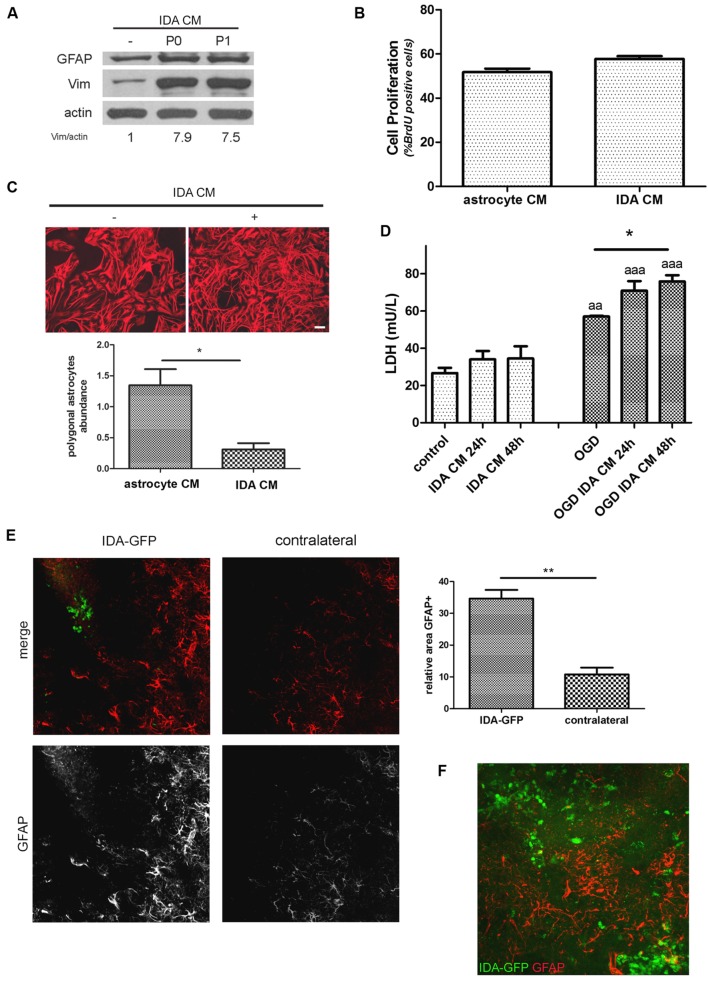
**Ischemia-derived astrocyte induce reactive gliosis and neuronal death. (A)** Immunoblot showing the increase in GFAP and vimentin (Vim) expression in primary cultured astrocytes incubated during 24 h with astrocytic control medium or CM of P0 (initial confluent cultures) or P1 (after one passage) IDA. Actin was used as loading control. Values indicate the OD ratio of vimentin vs. actin. **(B)** Quantitative analysis of cell proliferation (BrdU incorporation) after 24 h of incubation of primary astrocytes with IDA conditioned or control medium. The statistical significance was analyzed by Mann Whitney two tailed *t*-test. **(C)** Representative images of astrocytic stellation induced by the exposure of primary quiescent astrocytes to the CM from IDA cultures. Note the change from the astroglial resting polygonal phenotype to the reactive stellated morphology induced by the IDA CM, bar = 25 μm. Quantitative analysis of the abundance of polygonal astrocytes (number of polygonal cells per 1000 μm^2^) in the culture treated with IDA CM. The statistical significance was analyzed by Student two tailed *t*-test (*p < 0.05). **(D)** Primary cortical neuronal death analyzed by the LDH release (mU/L) into the culture medium after 24 or 48 h exposure to CM obtained from primary astrocytes or IDA. OGD exposure was performed during 30 min. Results were shown as mean ± SEM (*p < 0.05 as indicated; ^aa^*p* < 0.01; ^aaa^*p* < 0.001 vs. similar conditions in control neuronal culture after ANOVA and Student Newman Keuls post-test). **(E)** Low magnification confocal images showing the injection of eGFP-IDA in an uninjured brain. GFAP staining showing astrocytes is depicted in red; contralateral hemisphere injected with vehicle is presented as control. The graph shows the quantitative analysis of the area occupied by GFAP astrocytes in the IDA-injected hemisphere and the contralateral hemisphere. Statistical significance was tested by two tailed Student’s *t*-test (**p < 0.01). **(F)** Confocal image showing the interaction between injected IDA and resident astrocytes from the host animal.

When CM from IDA was collected and applied to quiescent primary astrocytes cultures, they showed an increased expression of the cytoskeletal proteins vimentin and GFAP (**Figure 4A**), and increased stellation (**Figure 4C**), both signs of reactive gliosis *in vitro*. However, IDA CM did not increase primary astrocyte cell division rate, as shown by BrdU incorporation (**Figure 4B**). Having in mind that neurons can be dramatically affected by soluble mediators secreted by astrocytes, we then tested the IDA CM effects on primary cortical neurons. While IDA CM had no significant effect on neuronal death of cortical neurons in control conditions, it increased LDH release when applied on OGD-exposed neurons, an *in vitro* manipulation that mimics the ischemic environment faced by neurons *in vivo* (**Figure 4D**).

To further test the ability of IDA to induce reactive gliosis *in vivo*, we obtained IDA from the eGFP+ rat transgenic strain [Wistar-TgN(CAG-GFP)184ys] subjected to CD ischemia (3 DPL) and, after 25 DIV, we injected the eGFP-IDA intracortically in *naïve* rats. As shown in **Figure 4E**, IDA induced a focal reactive gliosis in the injection site that was significantly larger compared with the contralateral hemisphere injected with vehicle (**Figure 4**). The injected eGFP-IDA migrated around the injection site but persisting in the area (**Figure 4F**). All these facts indicate that IDA can induce astroglial conversion to the reactive phenotype *in vitro* and *in vivo* and facilitate neurodegeneration of OGD-exposed neurons.

### IDA and Their Interaction with the *In Vitro* Glial Scar

**FIGURE 5:**
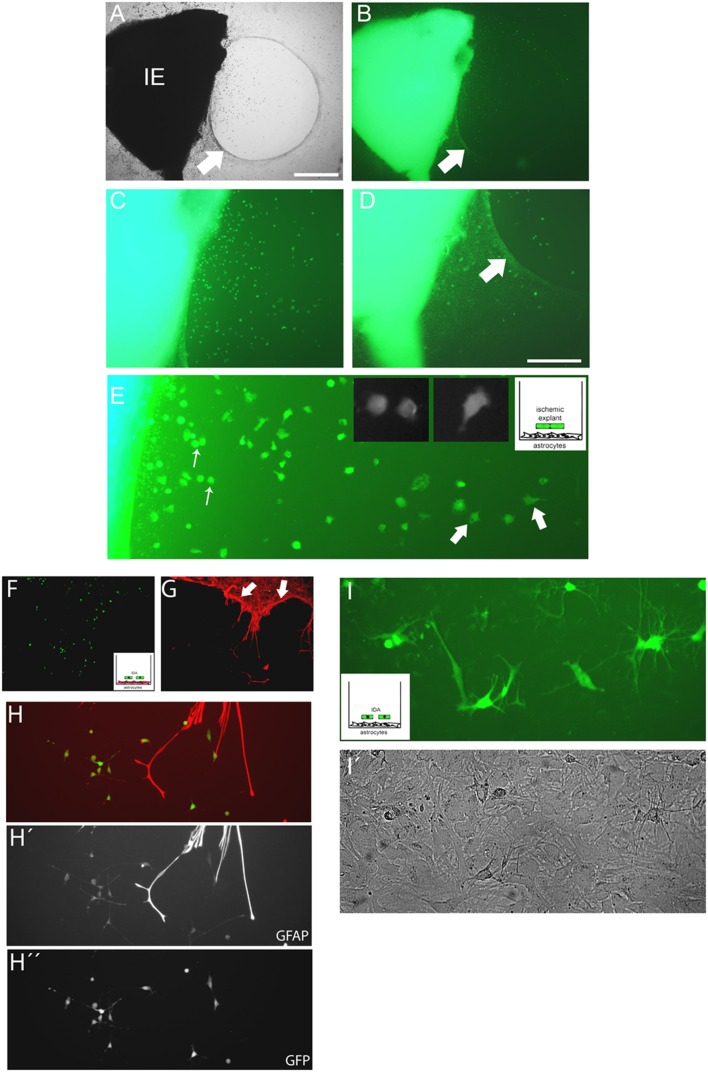
**Cells escaping from the ischemic explants induce glial scar formation *in vitro*, and IDA colonize the cell-free areas. (A,B)** Brain cortical ischemic explants (IE) obtained from eGFP+ animals subjected to ischemia after 3 DPL, seeded on primary astrocytic confluent monolayers during 3 DIV induced the formation of glial scar-like structures (arrow) that clustered and segregated cells escaping from explants, as easily seen in bright field **(A)** and escaping eGFP+ cells **(B)** observed under UV illumination, bar = 500 μm. **(C,D)** Representative images of cells escaping from ischemic explants showing that the majority of cells were clustered in the astrocytic-free areas **(C)** compared with the area with astrocytes limited by the scar-like structure (arrow) **(D)**. **(E)** High magnification detail of eGFP cells escaping from ischemic explants showing the different phenotypes, going from macrophage-like rounded phenotype (thin arrow) to the polygonal fusiform IDA (thick arrow). **(F,G)** IDA growing within the limits imposed by the corral glial scar-like structure by 5 days after seeding the eGFP+ ischemic explants on primary astrocytic monolayers. The mesh-like *in vitro* scar is clearly seen when stained with GFAP (red; arrow). **(H,H′,H**′′**)** Images of eGFP-IDA differentiated to stellated GFAP+ astrocytes in the vicinity of the astrocytic monolayer and scar-like structures stained with GFAP (red). **(I)** Several eGFP-IDA morphologically differentiated to stellated astrocytes **(I)** when seeded on top of a confluent primary astrocytic monolayer that is visible under phase contrast **(I**′**)**.

The close observation of cells escaping from ischemic explants showed that macrophage/microglial cells and IDA escape, but that IDA rapidly propagate and colonize the area. These observations imply that IDA are not repelled by macrophages as was described for primary astrocytes (Wanner et al., 2008, 2013). To further analyze the IDA characteristics, we incubated the 3 DPL ischemic explants dissected from ischemic cortical areas of the eGFP+ transgenic rat on top of a confluent monolayer of quiescent primary astrocytes, and then followed the co-culture by time-lapse microscopy to study the spontaneous interaction between the cells. As shown in **Figure 5A**, 3 DPL ischemic explants induced the retraction of primary astrocytes and the formation of a dense mesh with a scar-like structure that corrals cells escaping from the ischemic explants after 2 DIV of co-culture (**Figures 5A,B**), in close correlation with the effect observed with meningeal macrophages by Wanner et al. (2013). A detailed observation evidenced that a higher density of escaped ischemia-derived eGFP+ cells with the macrophage/microglial phenotype appeared in the astrocyte-free areas limited by the glial scar (**Figures 5C,D**). **Figure 5E** shows the macrophage/microglia cells and IDA colocalized in the astrocytic-free areas. Extending the co-culture time to 5 DIV showed that polygonal fusiform IDA turned into a classical stellated astrocytic morphology with increased GFAP expression, starting to colonize the area limited by the *in vitro* formed glial scar (**Figures 5F–H**). We conclude that IDA migrate, proliferate and differentiate in the areas delimited by the *in vitro* glial scar, showing a largely different response compared to primary astrocytes.

We then considered whether IDA could induce glial scar formation *per se*. For that purpose we cultured eGFP-IDA for very long term (25 DIV) and obtained an essentially pure IDA population as defined by their S100B^high^/GFAP^low^ immunolabeling and polygonal fusiform cell morphology. These IDA cultures were then dissociated and seeded on top of a primary astroglial cultures to follow the evolution of the co-culture after 3 and 7 days by tracking the changes in the eGFP+ cell population. We did not observe glial scar formation *in vitro*, but indeed we noticed that eGFP-IDA differentiated in the culture to the classical stellated astroglial phenotype (**Figures 5I,I**′) without showing the expression of oligodendrocytic lineage markers like O4 (data not shown). We conclude that IDA have unique features that render them able to proliferate in the vicinity of macrophages, colonize the cell-free areas produced by the astroglial retraction and retain the ability of differentiating into astrocytes, but do not induce glial scar formation.

### Blockage of Gamma Secretases Facilitates IDA Differentiation into Astrocytes

**FIGURE 6:**
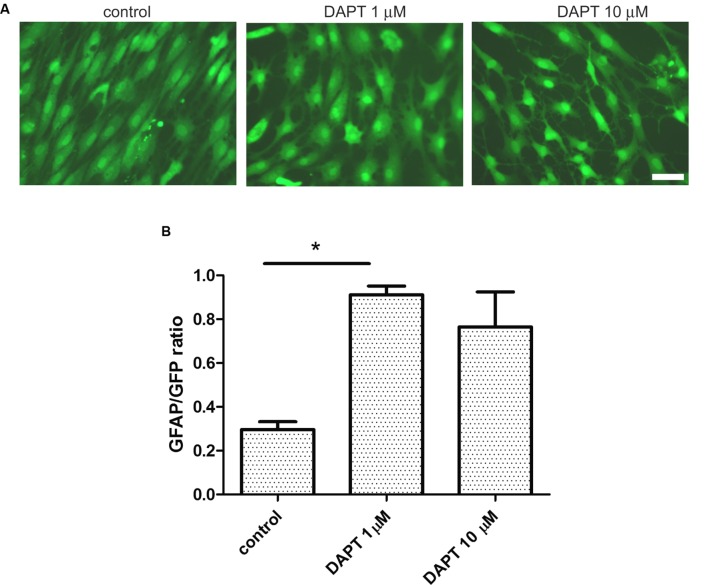
**Ischemia-derived astrocyte phenotype persistence requires gamma secretase activity. (A)** Representative images of eGFP-IDA cell cultures of 7 DIV showing the phenotypical transformation from the fusiform morphology to the stellated morphology induced by the 48 h-treatment with the gamma secretase inhibitor DAPT; control cells received vehicle; bar = 25 μm. **(B)** Relative abundance of GFAP immunostained astrocytes in the eGFP IDA cultures after treatment with different DAPT concentrations. Statistical significance was confirmed with the non-parametric Kruskal–Wallis test and Dunn’s Multiple Comparison Test (*p < 0.05).

Having observed that IDA seeded on top of a confluent primary astrocyte monolayer differentiate into astrocytes, we wondered which were the signals of the microenvironment that maintained the IDA phenotype. As it has recently been reported, the Notch1 signaling pathway regulates reactive astrocyte proliferation after ischemia (LeComte et al., 2015). We thus tested the effects of Notch1 signaling blockage by the gamma secretase inhibitor DAPT on the IDA cultures. With that aim, we cultured eGFP-IDA for 7 DIV and then treated the culture with DAPT and follow the morphological changes. As shown in **Figure 6A**, 48 h treatment with DAPT induced a phenotypic change in the IDA from the fusiform to a stellated phenotype and increased the ratio of GFAP immunoreactive cells in the culture (**Figure 6B**). On the other hand, blockage of NF-κB signaling dramatically reduced IDA survival and differentiation to astrocytes (data not shown). We conclude that gamma secretase activity is necessary to maintain the IDA phenotype, probably due to the requirement of a Notch1-dependent pathway.

## Discussion

Clinical observations have shown that, in the days following focal brain ischemia, the initial discrete necrotic core can propagate into the penumbra, thus worsening clinical prognosis. These clinical findings are also supported by data from experimental stroke models where neuronal death and reactive gliosis originally present in the core and vicinity may slowly progress toward the penumbra and adjacent healthy tissue, thus increasing the secondary damage. After variable periods of time, a dense mesh of intermingled fibrillar astrocytes known as the glial scar surrounds and isolates the ischemic region that has been occupied by fibromeningeal cells and blood derived myeloid immune cells.

A long standing hypothesis in the field is that, beneath a common immunocytochemical pattern and similar morphology, astroglial population is heterogeneous and there might be several masked reactive astrocytic phenotypes with different potential and physiological roles, including their ability to facilitate neuronal survival or death (Zhang and Barres, 2010; Anderson et al., 2014). In previous reports, different populations have been amplified by using extensive tissue culture manipulations including high levels of serum or other trophic factors (Yokoyama et al., 2006; Shimada et al., 2012; Götz et al., 2015). Consistently, in this study we have found and identified a subpopulation of astrocytes (IDA) that is induced in an early time window following focal brain ischemia (3–7 days) and, due to their increased migratory ability, spontaneously escape from ischemic tissue explants *in vitro*. Moreover, IDA can be isolated, maintained and amplified *in vitro*, have increased migratory ability, high cell division rate, express markers related to immature glial cell lineage and show reduced replicative senescence compared to primary astrocytic cultures. Interestingly, the CM obtained from IDA cultures induces reactive gliosis when applied on quiescent primary glia and facilitates neuronal death of OGD-exposed cortical neurons. When IDA were reintroduced into non-ischemic brains, they induced reactive gliosis *in vivo*. In addition, IDA rapidly proliferate and differentiate to the astroglial lineage, occupying the corral-like structures produced by the glial scar formation *in vitro*. While IDA expansion is facilitated by CM from OGD-stressed neurons or IDA cultures, the IDA phenotype is lost when gamma secretases are blocked.

It has been reported in experimental spinal cord injury lesions that focal injury induces the proliferation of reactive astrocytes that are heterogeneous in phenotype in a manner that varies with the distance from the lesion (Wanner et al., 2013). We have found here that IDA can only be amplified from tissue obtained from the ischemic core and penumbra in a time window between 3 and 7 days after the ischemic injury. Interestingly, it is not enough to induce a generalized reactive gliosis with peripheral LPS administration, since IDA were not obtained from the cortical explants of LPS-treated animals. Neither were IDA obtained from the contralateral hemisphere in animals subjected to unilateral ischemia by CD nor in sham animals. In our experiments, nestin expression in the focal ischemic area strongly correlates with the time window for IDA migration and isolation. Nestin is expressed by glial progenitors and also by reactive astrocytes induced by brain ischemia, but it is not expressed after peripheral LPS administration (Zamanian et al., 2012). We have found that the peak of nestin expression after ischemia by CD occurs between 3 and 7 DPL and IDA can only be isolated during this time window. By 14 DPL, when the glial scar is formed, IDA can no longer be obtained. Apparently, there is a time-window after the ischemic lesion which provides a permissive environment for IDA to proliferate and migrate. Furthermore, IDA could not be obtained either at very early time points such as 1 DPL, thus indicating that initial expansion of this atypical glial cell population probably requires additional signals beyond the acute lack of glucose and oxygen induced by brain ischemia.

Ischemia-derived astrocytes localized in the ischemic tissue spontaneously migrate away from the explants seeded in tissue culture chambers. Escaped IDA seem not to be repelled by Iba-1(+) macrophage/microglia. Again, this is an IDA unique feature since it has been shown that primary astrocytes spontaneously surround and segregate from macrophages and fibromeningeal cells into separated cell clusters (Wanner et al., 2008, 2013). IDA seem not to behave as primary astrocytes and can coexist in the area intermingled with macrophages.

The stable expression of S100B and low GFAP levels are other intriguing characteristics of IDA. In addition, in our hands IDA do not express detectable levels of NG2 (data not shown). Indeed, IDA are recovered from ischemic tissue before the time when NG2 positive cells appear in focal lesions (Cregg et al., 2014). When seeded on top of confluent primary astrocytic monolayers or in presence of DAPT gamma secretase inhibitor, a known blocker of Notch1 pathway (LeComte et al., 2015), IDA mainly differentiate into stellated astrocytes expressing GFAP without noticeable differentiation to oligodendrocytes, showing that they are restricted to the astroglial lineage.

Cell division rates and migratory abilities of IDA after *in vitro* isolation are remarkable. The IDA high proliferation rate allows the rapid colonization of the tissue culture plates, even starting from a highly diluted culture. We show here that this process is facilitated by soluble mediators secreted by OGD exposed neurons or by medium from the IDA culture itself, thus evidencing a major role of soluble mediators from the microenvironment in IDA expansion. Indeed, IDA also showed reduced replicative senescence, permitting long term cultures and several cell passages without substantial loss of cell viability or change in the S100B^high^/GFAP^low^ phenotype. Both the high proliferation rate and lack of replicative senescence are shared by the ALS AbAs described by Díaz-Amarilla et al. (2011) in the lesions of SOD1^G93A^ mice. On the other hand, IDA seem to retain the ability to differentiate into mature astrocytes, thus evidencing a partially more restrictive lineage when compared to the cells forming the neurospheres obtained after ischemia by Shimada et al. (2012). Rat primary microglia expressing NG2 can give rise to neurospheres that originate neuroectodermal cells in a two-step culture using 10 and 70% serum-supplemented medium (Yokoyama et al., 2006). However, IDA do not seem to derive from NG2 cells. Our experiments with eGFP-BMMC evidenced that IDA isolated from ischemic brains are not derived from immune myeloid precursors recruited to the ischemic injury site, neither do they express NG2. Shimada et al. (2012) were able to obtain neural spheres in cultures derived from the cortical peri-infarct area that are supposed to be originated in reactive astrocytes since they are tagged with GFAP-tdRFP. However, these glial-derived neurospheres require EGF and FGF to be developed and expanded *in vitro*, showing an striking contrast with IDA, which spontaneously escape from ischemic tissue explants, migrate and proliferate at a high division rate. The IDA obtained by us from tissue explants proliferate *in vitro* in normal astroglial culture medium as a monolayer without any added trophic factor beyond the 10% classical level of FCS. Reactive astrocytes are heterogeneous after injury as they differentially express proteins associated with stem/progenitor cells (White et al., 2010). We cannot rule out, however, that IDA could be further de-differentiated and form neurospheres, given the necessary specific supportive trophic factors. Since migration of SVZ neuroblasts was not detected by 3 days after stroke (Shimada et al., 2012), we propose that IDA are being generated from local precursors activated by the permissive environment generated by the ischemic injury. Another distinctive characteristic of IDA is the ability to induce reactive gliosis on quiescent astrocytes *in vitro* and *in vivo*. Exposure to IDA CM also facilitates neuronal death in OGD-exposed primary cortical neurons. Interestingly, only neurons that have been exposed to OGD are significantly sensitive to the IDA toxic effects, thus supporting the idea that only stressed neurons can be killed by IDA. Both us and others have reported that OGD induces the expression of Pattern Recognition Receptors as RAGE, that are likely to be involved in this astrocyte-mediated neuronal killing (Villarreal et al., 2011; Kamide et al., 2012; Angelo et al., 2014). Furthermore, AbAs in the ALS transgenic model showed the ability to specifically induce motor neuronal death without affecting other neuronal types (Díaz-Amarilla et al., 2011).

Glial scar formation is a key feature of focal CNS lesions. As previously demonstrated by Wanner et al. (2008, 2013), primary astrocytes actively surround macrophages and spontaneously form scar-like structures that corral inflammatory meningeal macrophages in a STAT-3 dependent manner. By using a similar experimental paradigm, we incubated ischemic explants on top of confluent primary astrocytes and observed that astrocytes actively form corral structures surrounding the explants. IDA migrate from the ischemic tissue explants occupying the corral structure formed by primary astrocytes. It is clear that IDA are not being repelled by macrophages and can proliferate and differentiate in their presence. IDA are not able, however, of forming scar-like structures *in vitro* or *in vivo* as shown by the IDA-astrocytes co-culture and *in vivo* intracortical cell transfer. In this scenario, a tempting hypothesis is that IDA expansion and proliferation is the result of the presence of soluble mediators from the ischemic tissue that activate local resident astroglial cell precursors. Stabilization of the IDA phenotype, however, seems to require gamma secretase activity and thus Notch1 signaling is an obvious candidate for that role. Based on the results obtained in the inducible transgenic GFAP-CreER-Notch1-cKO and GFAPCreER-ETBR-cKO mice, LeComte et al. (2015) determined that the Notch1–STAT3–ETBR axis connects a signaling network that promotes reactive astrocyte proliferation after brain ischemia. In this scenario, it is tempting to speculate that IDA phenotype stabilization requires Notch1 signaling, and thus the gamma secretase inhibitor DAPT, that blocks Notch1 signaling, allows IDA differentiation into GFAP-expressing astrocytes. However, the fully testing of this pathway on IDA deserves further experimentation.

In summary, this study reports the identification and isolation of an atypical astrocyte sub-population, named IDA, induced by brain ischemia and presumably localized in the ischemic penumbra. The most striking characteristics of this cell type include the facilitation of neuronal death of OGD-stressed neurons and the induction of reactive gliosis on quiescent astrocytes. In view of our results, we consider IDA to have a key role in the pathophysiological features of brain ischemia, specifically in the progression of reactive gliosis, neuronal death and subsequent proliferation within the limits of the glial scar, thus representing an interesting target for therapeutic strategies.

## Author Contributions

AV and GR equally contributed to this work. AV, VM, PS-A, LB, and AR designed the experiments; AV, GR, VM, VC, VU, and MD-T did the experimental work; AV, VM, GR, LB, and AR analyzed and interpreted the data; PS, LB, and AR wrote and revised the article.

## Conflict of Interest Statement

The authors declare that the research was conducted in the absence of any commercial or financial relationships that could be construed as a potential conflict of interest.

## Supplementary Material

FIGURE S1 **(A)** Most IDA escaping from ischemic explants express S100B and only some of them are differentiated into stellated GFAP+ astrocytes after 7 DIV, bar = 30 μM. **(B)** Representative images of eGFP+ cells found in blood smear, liver and brain in the ischemic animals after 3 DPL. The insets show high magnification images of eGFP+ cells present in these preparations.

FIGURE S2 **Cell recovery after ischemia. (A)** Total number of cells recovered at 1 or 7 DIV starting from ischemic cortex of 1, 3, or 7 DPL. **(B)** Percentage of microglia/macrophage phenotype after 1 or 7 DIV starting from similar ischemic cortical tissue of 1, 3, or 7 DPL. Values represent the mean ± SEM and statistical significance of the effects was confirmed with ANOVA and Student Newman Keuls post-test (**p* < 0.05; ***p* < 0.01).

FIGURE S3 **Images show the persistent stable polygonal fusiform phenotype of IDA after none (P0), one (P1), or two (P2) passages, bar = 15 μM**.
