# Supplementary figures and images for: Isolation and Characterization of Ischemia-Derived Astrocytes (IDAs) with Ability to Transactivate Quiescent Astrocytes
Source: Front Cell Neurosci. 2016 Jun 1;10:139. doi: 10.3389/fncel.2016.00139 (PMC4888624; doi:10.3389/fncel.2016.00139)

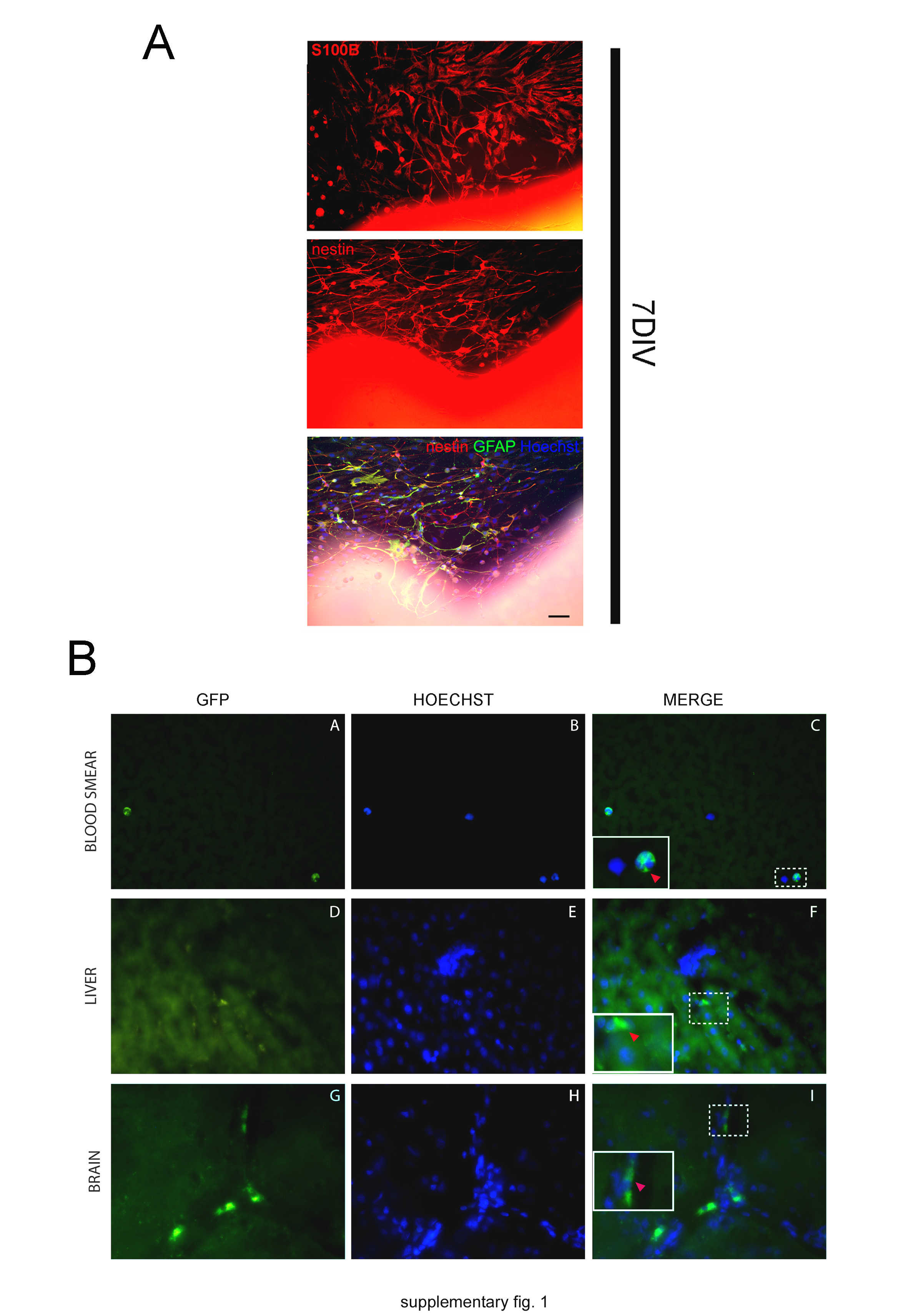

Supplement: FIGURE S1 — (A) Most IDA escaping from ischemic explants express S100B and only some of them are differentiated into stellated GFAP+ astrocytes after 7 DIV, bar = 30 μM. (B) Representative images of eGFP+ cells found in blood smear, liver and brain in the ischemic animals after 3 DPL. The insets show high magnification images of eGFP+ cells present in these preparations. [file Image_1.TIF]

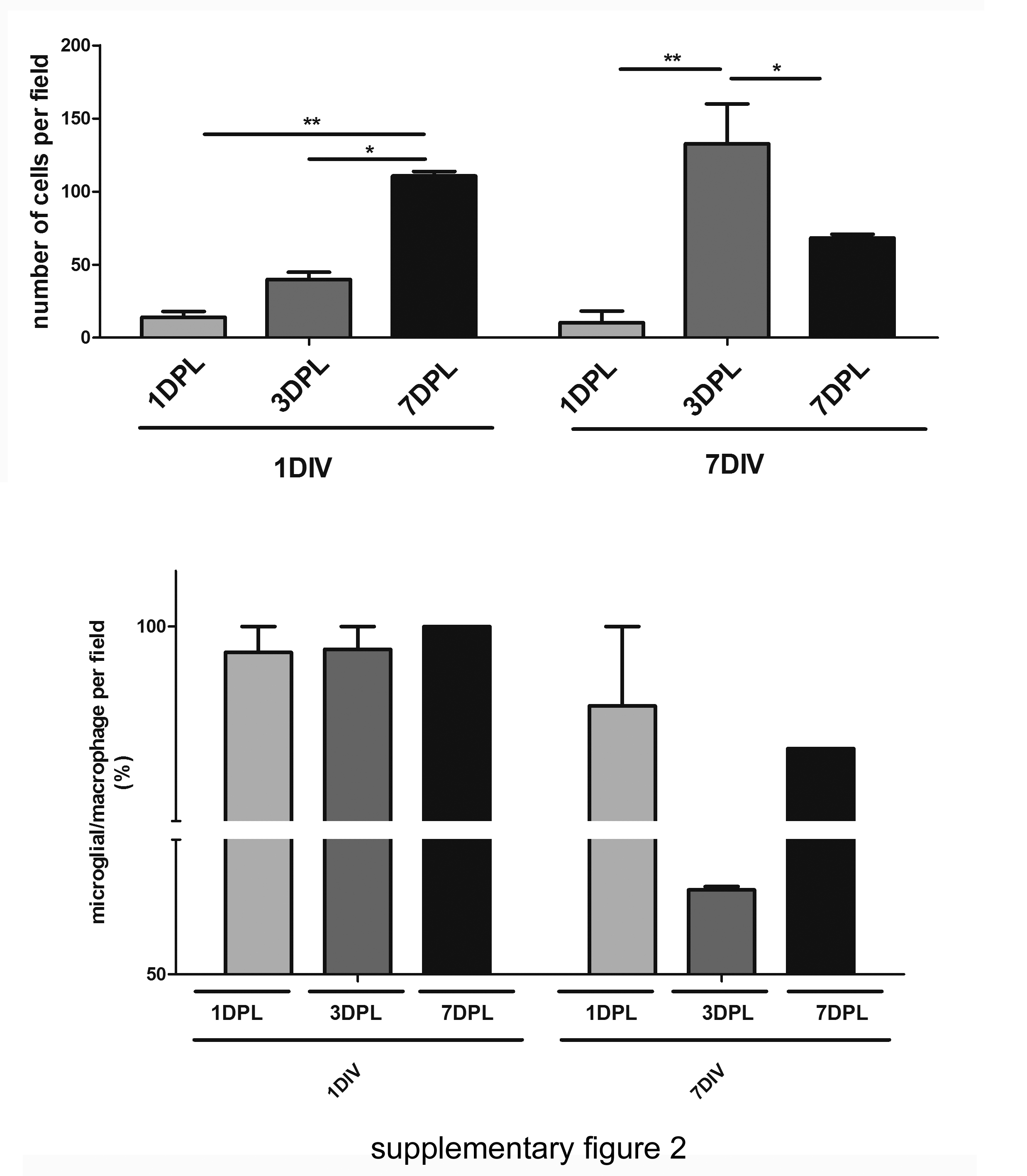

Supplement: FIGURE S2 — Cell recovery after ischemia. (A) Total number of cells recovered at 1 or 7 DIV starting from ischemic cortex of 1, 3, or 7 DPL. (B) Percentage of microglia/macrophage phenotype after 1 or 7 DIV starting from similar ischemic cortical tissue of 1, 3, or 7 DPL. Values represent the mean ± SEM and statistical significance of the effects was confirmed with ANOVA and Student Newman Keuls post-test (*p < 0.05; **p < 0.01). [file Image_2.TIF]

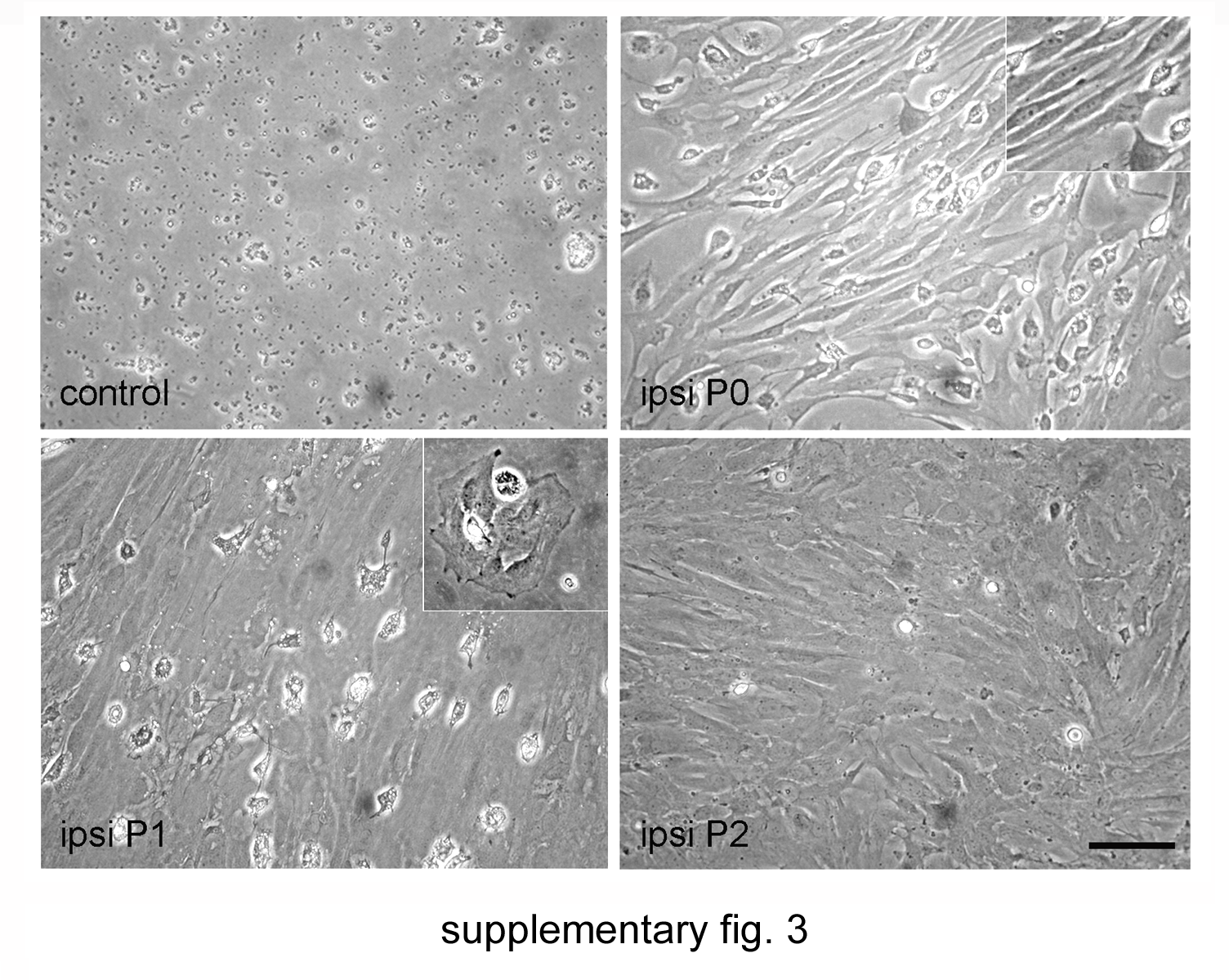

Supplement: FIGURE S3 — Images show the persistent stable polygonal fusiform phenotype of IDA after none (P0), one (P1), or two (P2) passages, bar = 15 μM. [file Image_3.TIF]
